# Supplementary material for: 3D Method for Occlusal Tooth Wear Assessment in Presence of Substantial Changes on Other Tooth Surfaces
Source: J Clin Med. 2020 Dec 4;9(12):3937. doi: 10.3390/jcm9123937 (PMC7761944; doi:10.3390/jcm9123937)
Supplement: Supplementary file 1 [file jcm-09-03937-s001.pdf]

3D Method for Occlusal Tooth Wear Assessment in Presence of Substantial Changes on Other Tooth Surfaces

Nikolaos Gkantidis*, Konstantinos Dritsas, Christos Katsaros, Demetrios Halazonetis and Yijin Ren

Supplementary Figures:

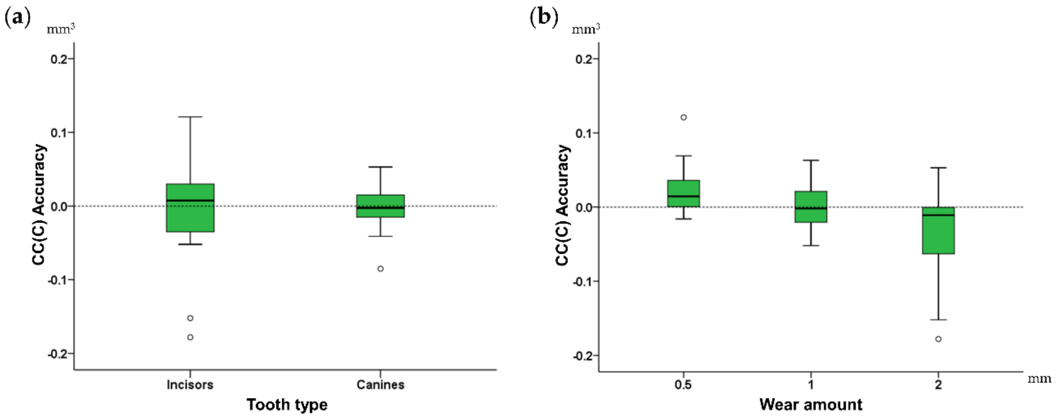

Figure S1. Box plots showing in the Y-axis the difference of the technique of choice (complete crown (CC), setting C) from the gold standard technique in tooth wear measurements (**a**) by tooth type and (**b**) by amount of tooth wear. The upper limit of the black line represents the maximum value, the lower limit the minimum value, the box the interquartile range, and the horizontal black line the median value (trueness). Outliers are shown as black circles with a step of 1.5×IQR (interquartile range). Zero value indicates perfect agreement with the gold standard (horizontal dashed line). The vertical length of each plot indicates precision.

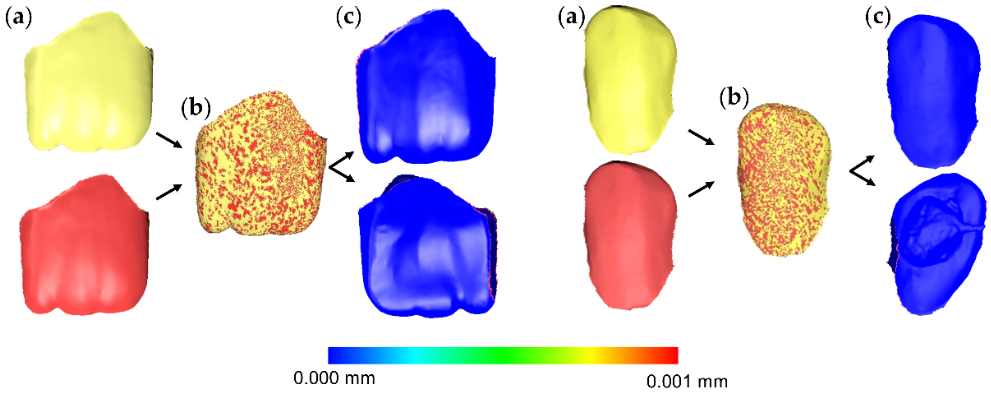

Figure S2. Superimposition of identical surface models of a maxillary incisor (left) and a mandibular canine (right). (**a**) Original tooth (yellow) and duplicate (red). (**b**) Superimposed tooth crowns using the complete crown technique and setting C (20% estimated overlap) shown from the buccal aspect. (**c**) Color coded distance maps of the superimposed crowns, shown from the buccal (above) and the palatal (below) side.

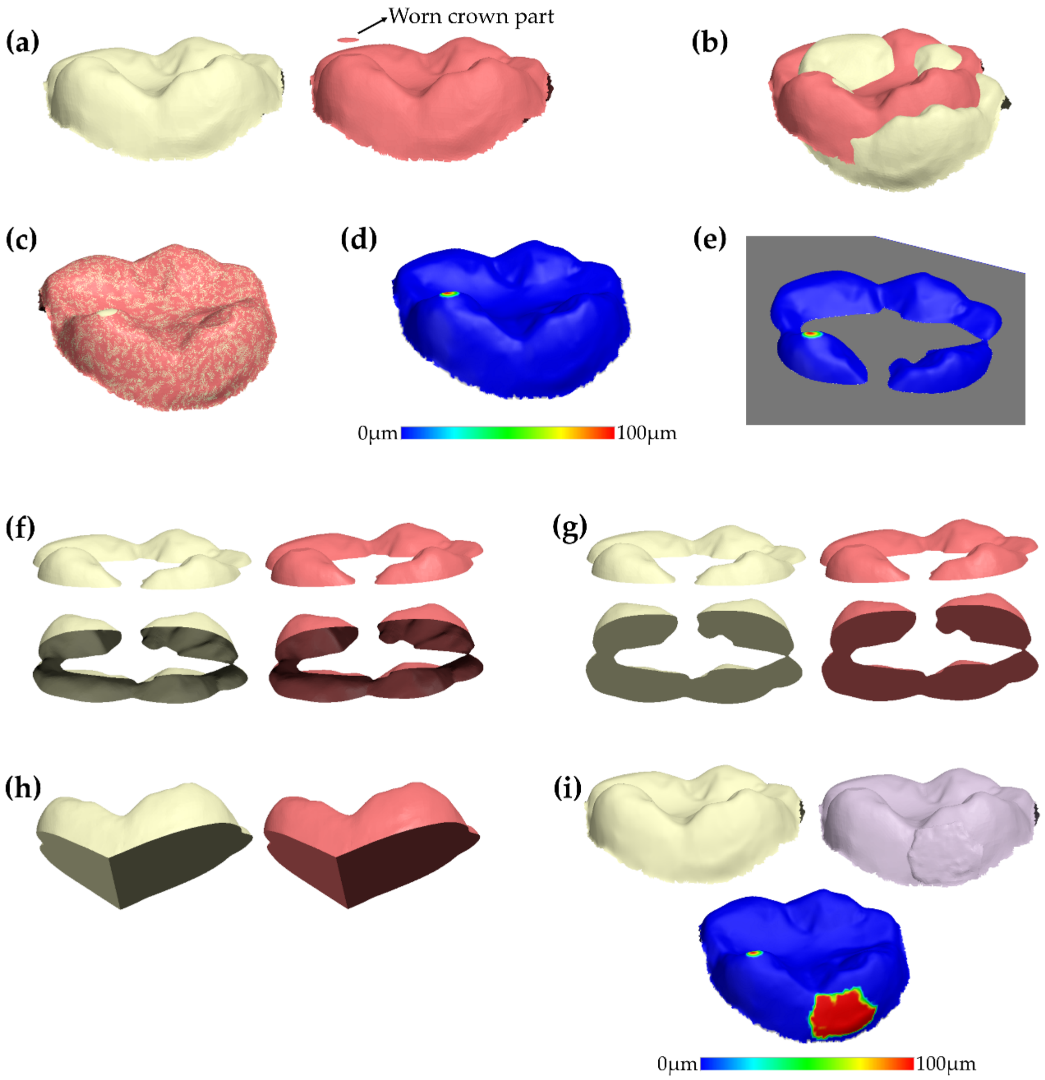

Figure S3. Tooth wear measurement in a mandibular molar with limited loss of tooth structure. (**a**) Tooth before (light yellow) and after (light red) tooth wear simulation. (**b**) Manually approximated tooth crowns. (**c**) Superimposed tooth crowns using the complete crown technique and setting C (20% estimated overlap). (**d**) Color coded distance map of the superimposed crowns. (**e**) Level (grey) used to simultaneously slice the two crowns. (**f**) Sliced tooth crowns. (**g**) Holes filled to create watertight models, and thus, calculate volumes. (**h**) Watertight models after slicing the same superimposed models using two slicing levels. (**i**) Tooth before (light yellow) and after (light purple) tooth wear plus retainer placement simulation and color coded distance map of the superimposed crowns using the complete crown technique and setting C (20% estimated overlap). The wear amount measured with one slicing level (**a**–**g**) was 0.021 μm^3. The wear amount measured with two slicing levels (**h**) was 0.020 μm^3. The wear amount measured following the retainer placement (**i**) was 0.020 μm^3. The wear amount measured with the gold standard technique and two slicing levels was 0.020 μm^3.
